# HOURS CAREGIVING, ADLS, ROLE OVERLOAD, AND DEPRESSION SYMPTOMS: AN EXAMINATION AMONG FAMILY DEMENTIA CAREGIVERS

**DOI:** 10.1093/geroni/igae098.2414

**Published:** 2024-12-31

**Authors:** Claire McDonald, Adrian Bravo

**Affiliations:** University of Maryland Baltimore County, Baltimore, Maryland, United States; William & Mary, Williamsburg, Virginia, United States

## Abstract

The present study examined the associations between hours spent caregiving, Activity of Daily Living (ADL) dependency, role overload as a stressor, and depression symptoms among caregivers of adults aged 65 and older living with probable dementia. Specifically, we hypothesized that more time spent caregiving and greater ADL dependency would both be associated with more depression symptoms via greater role overload. To test study aims, we analyzed data from round 11 of the National Health and Aging Trends Study (NHATS) and the National Study of Caregiving (NSOC). To replicate findings from NHATS and NSOC, a second sample of data was collected using Academic Prolific. In the NHATS sample, there were 283 caregivers and the majority of caregivers were female (70%), white (63%) and reported an average age of 65. For the Prolific sample, there were 150 caregivers and the majority were female (51%), white (68%), and reported an average age of 40. Mediation models were tested using the PROCESS Macro (Hayes, 2013) in SPSS. In both NHATs and Prolific samples, role overload significantly mediated the associations between hours caregiving and depression symptoms (NHATs indirect β =.119, CI [.07,.18]) (Prolific indirect β =.118, CI [.02,.21]). In both NHATs and Prolific samples, role overload significantly mediated the associations between ADL dependency and depression symptoms (NHATs indirect β =.07, CI [.01,.13]) (Prolific indirect β =.111, CI [.02,.20]). These findings highlight the impact of burden among family dementia caregivers and should be investigated in more diverse populations.

